# Conflict, epidemic and faith communities: church-state relations during the fight against Covid-19 in north-eastern DR Congo

**DOI:** 10.1186/s13031-022-00488-4

**Published:** 2022-11-09

**Authors:** Sadiki Kangamina, Jean-Benoit Falisse, Amuda Baba, Liz Grant, Nigel Pearson, Yossa Way, Emma Wild-Wood

**Affiliations:** 1 Université Anglicane du Congo, Bunia, Democratic Republic of the Congo; 2 Centre of African Studies, The University of Edinburgh, 15a George Square, Edinburgh, EH8 9LD UK; 3 Institut Supérieur de Techniques Médicales de Bunia (ISTM/Bunia), Bunia, Democratic Republic of the Congo; 4 Global Health Academy, The University of Edinburgh, Edinburgh, UK; 5 Oxford, UK; 6 Centre for the Study of World Christianity, The University of Edinburgh, Edinburgh, UK

**Keywords:** DRC, Religion, Conflict, Covid-19, Health system, Faith-based health provision, Ebola

## Abstract

**Background:**

Understanding and improving access to essential services in (post)-conflict settings requires paying particular attention to the actors who occupy the space left ‘empty’ by weak or deficient State institutions. Religious institutions often play a fundamental role among these actors and typically benefit from high trust capital, a rare resource in so-called ‘fragile’ states. While there is a literature looking at the role faith organisations play to mobilise and sensitise communities during emergencies, our focus is on a different dimension: the reconfiguration of the relationship between religion and health authorities impelled by health crises.

**Methods:**

We analyse observations, interviews, and focus group discussions with 21 leaders from eight different religious groups in Ituri province in 2020–2021.

**Results:**

Faith institutions handled the Covid-19 lockdown period by using and redeploying structures at the grassroots level but also by responding to health authorities’ call for support. New actors usually not associated with the health system, such as revivalist churches, became involved. The interviewed religious leaders, especially those whose congregations were not previously involved in healthcare provision, felt that they were doing a favour to the State and the health authorities by engaging in community-level awareness-raising, but also, crucially, by ‘depoliticising’ Covid-19 through their public commitment against Covid-19 and work with the authorities in a context where the public response to epidemics has been highly contentious in recent years (particularly during the Ebola outbreak). The closure of places of worship during the lockdown shocked all faith leaders but, ultimately, most were inclined to follow and support health authorities. Such experience was, however, often one of frustration and of feeling unheard.

**Conclusion:**

In the short run, depoliticization may help address health emergencies, but in the longer run and in the absence of a credible space for discussion, it may affect the constructive criticism of health system responses and health system strengthening. The faith leaders are putting forward the desire for a relationship that is not just subordination of the religious to the imperatives of health care but a dialogue that allows the experiences of the faithful in conflict zones to be brought to the fore.

## Introduction

Understanding and improving access to essential services in conflict and post-conflict settings requires paying particular attention to the actors who occupy the space left ‘empty’ by weak or deficient State institutions [1]. Among these actors, religious groups often play a fundamental role in providing care [2] and raising awareness of health issues [3]. Their role is even more critical during humanitarian crises, including epidemics [4, 5], not least because populations living in so-called fragile’ settings typically trust religious actors more than State actors [6, 7]. Yet the role of religious denominations in contexts marked by both the Covid-19 pandemic and armed conflict has received only limited attention [8]. Many frameworks developed to address Covid-19 in conflict settings barely mention religious communities and institutions [9] and the few that do tend to limit their role to community mobilisation [10]. The present article seeks to go beyond such oft-documented instrumentalization of churches for public health purposes. It discusses a more fundamental and general dynamic: the relationship between religious denominations, the State, and health authorities in a fragile context marked by a general mobilisation to fight an epidemic. How did religious denominations experience the Covid-19 pandemic and the State-led response? What does it tell us about the relation between religious groups and health authority relations in so-called ‘fragile’ contexts?

Our focus is on faith communities in the city of Bunia and the town of Kasenyi, in the north-eastern Ituri province of the Democratic Republic of Congo (DRC) and, to a lesser extent, the town of Oicha in the north of the neighbouring province of North Kivu. The region is one of the most troubled in the country, at least since the end of the brutal regime of President Mobutu and the first Congo war (1996–1997): armed groups abound and have committed numerous abuses against the civilian population, including massacring, looting, and sacking villages [11]. The repeated cycles of Ebola, cholera, and bubonic plague epidemics, and more recently, Covid-19, have compounded the crises. The Catholic Church and Protestant churches manage many health facilities and even have a predominant place in managing certain health districts. Our article shows how the Covid-19 measures pushed other denominational actors typically more distant from the health system—revivalist churches and Islamic communities, among others– to become involved in healthcare. The experience is often marred with frustration.

The following section presents the context of Ituri and the debates around the involvement of religious denominations in health. We then introduce our methods, which are based mainly on interviews with key informants and field observations, before presenting our main findings.

## Context

### Security, health and religious situation in Ituri

Eastern DRC has experienced significant instability and repeated violence for over 25 years; more than 130 armed groups were reported active in the region in 2019 [12], and socio-economic tensions crystallising around ethnic identities are rife. In May 2021, the President of the Republic declared a ‘state of siege’ (*état de siège*) and handed over the administration of the provinces of Ituri and North Kivu to the army. By the time of writing, it had not stopped violence. In June 2021, 1.7 million people were forcibly displaced in Ituri, almost half the province’s estimated population. The effects of the conflict on health are devastating and come after the local health system had to deal with a significant Ebola outbreak in 2019–2020 and a more recent bubonic plague outbreak. The Ebola response was heavily criticized by the population, with many people decrying what they termed the ‘Ebola business’ of the State and international aid agencies [13, 14]. It further eroded trust in state institutions [6].

Faith-based health facilities and the religious denominations that manage dispensaries, hospitals and sometimes even health zones on behalf of the Ministry of Health [15], provide front line services. This health service provision can be traced back to the colonial period. After independence, the collapse of the Zairian public services from the late 1970s onwards, combined with the structural adjustment policies and then finally the wars of the 1990s, contributed to an even greater focus on health services directly organised by non-profit organisations, including the churches, and the private sector.

Nationally, the population is estimated to be 95.1% Christian, 1.5% Muslim, and 2.5% Indigenous religions [16]. Among Christians, there is a growing number of followers of Pentecostal and so-called ‘revivalist’ churches [17] who profess faith healing. Muslims are a historical minority with a relatively stable membership. The Catholic Church, which has the largest membership, and the federation of Protestant churches (*Eglise du Christ au Congo*, Congo Christ Church, CCC) are also political forces; they have played a role in various key episodes in the country’s life, from the fall of Mobutu [18] to the most recent opposition to the appointment of the new president of the independent national electoral commission [19].

### The Covid-19 pandemic in the DRC

The Covid-19 pandemic has taken on two distinct faces in the DRC. On the one hand, the number of cases recorded is lower than in southern Africa (and in Europe and America), with 43,333 cases and 973 deaths recorded as of 7 July 2021 and, even though detection capacity is low, there has been no anecdotal reports of high excess mortality [20]. On the other hand, however, among a more affluent population and the urban ‘elite’, a different picture emerged: Covid-19 was reported as the cause of death of nearly 5% of the members of parliament (*députés*) in June 2021 [21]. The Congolese government responded by ‘recycling’ some of the mechanisms developed in the fight against Ebola, such as the Technical Secretariat of the Response and data sharing and analysis mechanisms [22] and taking measures similar to other countries, such as lockdowns (see Table 1). They have had clear adverse effects on social and economic life [23].

**Table 1:** Main measures against Covid-19 in the DRC. *Source*: Coronanet dataset  https://www.coronanet-project.org/

19 March–22 July 2020 16 February 2021- 14 February 2022	Prohibition of all gatherings, meetings, celebrations of more than 20 people in public places Restriction of all gatherings, meetings, celebrations of more than 100 people in public places
19 March–10 August 2020 20 January–15 February 2021	Closure of schools, universities and higher institutes.
19 March–22 July 2020	Suspension of all worship
19 March–22 July 2020	Suspension of sports activities in stadiums and other sports venues
19 March–22 July 2020	Closing of discos, bars, cafés, terraces, and restaurants
19 March–22 July 2020	Prohibition of mourning in houses.

## Methods

This article is part of an interdisciplinary project involving social sciences, public health, and religious studies about faith communities during the Covid-19 crisis in Ituri and the north of North Kivu province. It is complemented by two other papers focussing on (1) faith community members’ conceptions of Covid-19, its origins, and its meaning [24] and (2) faith community engagement with public health messages about Covid-19 [25].

Given the health and security situation, most data collection was done closely with researchers linked to the Université Anglicane du Congo and living in Bunia, Oicha, and Kasenyi. This community base was an asset in reaching respondents but may also lead to potential biases (see below). We distinguish three types of data collection: (1) observation of the health and security situation during the period from March 2020 to June 2021; (2) semi-structured qualitative interviews conducted directly face-to-face with 21 religious leaders (interviews, see Table 2), health officials (4 interviews in the Bunia health zone), and members of faith communities (3 focus groups). Most interviews were conducted in December 2020 and February 2021 (with additional interviews with the general population in July 2021). These more in-depth interviews form the core of our analysis; and (3) a discussion of our preliminary findings with 11 religious leaders (Catholic, Evangelical, Anglican, Muslim, Pentecostal, Revivalist) on 29 June 2021.

**Table 2:** Religious communities covered by the study (with number of interviews)

Communities that manage clinics and hospitals	Communities that do not manage clinics and hospitals
Roman Catholic Church (2)	African and independent churches (Obumu, Chrisco, Combat Spirituel, Independent)
Anglican Church (2)	Revival churches (3)
Sunni mosques (3)	Lam-Te-Kwaro (ancestor worship)
CECA 20 (protestant) : Communauté Évangélique au Centre de l’Afrique (3)	
Other Protestant churches (2)	

There exists no precise census of religious denominations in our research area. The selection of religious leaders is representative of the denominations most visible in the public space ; it includes denominations that have clinics and those that do not.

The thematic analysis presented below is based primarily on the transcripts of the interviews and workshop proceedings. Passages dealing with the relationship between faith communities (or religion more generally) were systematically identified and then further divided into sub-categories according to the theme. The categories and sub-categories correspond to the sub-sections of the results section. We indicate either in the text or in square brackets the affiliation of the people quoted and the source (interview or workshop). Research participants have agreed to be identifiable.

## Main findings

We present our findings in three parts. Our interviews, workshop, and observations highlight the sense of shock provoked by the mandatory closure of places of worship—a corollary of the lockdown measure. We describe how lockdown was experienced and rationalised by religious leaders and communities; how ‘coping strategies’ were put in place by religious groups, and, finally, how the relationship between religious groups and health authorities (and the state) has been affected by the pandemic experience.

### The closure of places of worship

The obligation to close places of worship during the first lockdown was unexpected for all religious leaders and their communities. The Anglican Diocesan Medical Service called it “a big surprise because it has not happened for years”. Although political and administrative authorities had sometimes coerced specific) churches to close in the recent past, the compulsory closure of *all* places of worship had not happened before.

The closure generated substantial anxiety among religious communities, especially those whose liturgy and prophetic preaching emphasised the “end times” of the world because of sin. Our data also showed religious leaders who are aware that they are subject to the temporal power of the government and that opposing the closure of places of worship is not an option. The leader of an independent revivalist church in Bunia, the *Ministère Chrétien du Combat Spiritual* (the *Christian Ministry of Spiritual Combat*), explained that:

“when the number one in the country says something, we must only submit. We submitted to the demands of the President; everyone prayed at home, everyone was at home.“

Other feelings emerged after initial surprise and fear, ranging from acceptance to outright indignation. Feelings were not consistent along denominational lines. At one end of the spectrum, some leaders described closures as excessive and potentially counterproductive. Their resentment drew on three elements: (1) the feeling that the closure of places of worship was disproportionate; (2) the suspicion that the government’s response was inconsistent, and in particular the fact that if places of worship were to be close then markets should also be closed; and (3) the strong sense that the closure was an attack on the power and value of the Christian faith. A workshop participant, a Catholic priest, explained the reasoning of many of the leaders we met:

“When the church is closed, it is the church of God that we pray to [that is closed]. It is God who is above all, and he is the one who can help us fight this scourge, but we are forbidden even to pray...“

Other religious leaders, while regretting the closure of places of worship, accepted the necessity of the action and often rationalised, as the Rehobot church (revivalist) did, that “lockdown cannot affect the Christian life of a Christian [because there is still] family prayer and personal prayer time”. Covid-19 was sometimes even presented as an opportunity by some revivalist church leaders—one of them explained that “in our church, we advise people to make a radical confession” and that the Covid-19 lockdown effectively created conditions more conducive to such confession.

In many respects, it is respect for State authority that best explain compliance with the order to close places of worship. The acceptance of the rationale of the government’s decision general came much after the closure order, if at all, and seemed related to witnessing Covid-19 cases in the community. This contact with the disease was, however, not always enough and in a complementary article [24] we show how doubts about the virus’ biomedical nature endured.

The decision not to close churches during the second lockdown was met with relief by many religious leaders, although some suggested that the closure of schools but not places of worship signalled incoherence in public action:

### Maintaining faith community during lockdown

Faced with a strict lockdown order, many religious leaders spoke of the need to “continue with their pastoral mission”. Many churches adapted to the challenges. For example, some mobilised or created “many small cells [informal gathering of community members for support, bible study, and prayer] throughout the city” [interview, CECA 20, 18]. They were active before the pandemic in the case of the Catholic and some Protestant churches, such as CECA-20, but most denominations soon followed suit. These cells, which are small enough to operate within the Covid-19 rules or work remotely, maintained religious life during the lockdown. They also took an active role in Covid-19 sensitisation, often basing their action on religious ideas, since, as a cell leader explained: “our pastoral mission is also an educational mission”. Importantly, in a few instances the cells helped collect the offerings that pay for the church’s running costs and its leaders’ salaries (an issue discussed in more details in a recent cross-country study, see [26]). Lockdown disrupted the economic model of churches dependent on offerings from the faithful during worship, but soon offerings were given to cell leaders, who passed them on to church leaders. Other mechanisms, such as donation boxes placed outside churches or even offerings via mobile money, were also implemented. In some groups (Protestants (CCC), Catholics, and Muslims), community radios were used to encourage offerings (or tithes and thanksgivings) and keep in touch with the congregation and broadcast awareness messages about Covid-19. Social media appeared in our research but was cited as a nuisance—a source of ‘fake news’ that is difficult to combat– more than a useful channel used by churches.

In practice, the army and police did come and check that places of worship were not being used, and our research found no evidence of meetings taking place. Nevertheless, these actions reinforced the idea of churches being coerced, as explained by a representative of an independent church:

“people from the security forces started to chase people, it’s like the church leaders have sinned [...] so I think with this, the church leaders just ‘consumed’ [*consommé*, ‘bit the bullet’ in this case] and accepted, complied with what the government had decreed”.

This element is essential and explains the ‘acceptance’ of the closure of places of worship described in the previous section and work with the government described in the next section. The idea of enduring—i.e. being subjected to the (health) authorities rather than feeling fully responsible and involved– is fundamental.

### A change in the relationship between the health system and religious denominations?

#### Business as usual

For the four denominations who also provide medical services—the Anglican Church through its Medical Service, the Catholic Church through Caritas, and to a lesser extent the CECA20 and Muslims– medical work in times of Covid-19 was a natural extension of their important role in the public health system in Ituri. Interviews with health officials confirm this impression: “[with Covid-19] nothing has changed regarding the church health structures” for an official in the Bunia health zone. Covid-19 is neither the biggest problem nor the most urgent one for these faith-based health facilities, and the tasks they are asked to perform are not controversial (contrary, for instance, to other issues such as family planning). In addition to this role, local leaders of these four faith groups explained that they sought to prevent and reduce the burden of Covid-19 beyond their own health facilities by working with their faith communities (for more details on this, see our article [25]). In practice, anti-Covid-19 messages are relayed by religious leaders but also often integrated into worship: “in every prayer, even if it is only for a few minutes, we tell our faithful about some of the measures decreed by the Congolese government, concerning Covid-19, and also practising it” [interview, Muslim, 1]. However, this did not happen without clashes; we have already explained the confusion in relation to the rationale behind Covid-19 measures, and a representative of the Catholic Church did not hesitate to report that he sometimes felt “betrayed by the government, which is supposed to make things clear to people”.

#### New actors

Religious leaders whose denominations do not organise biomedical services felt that, by complying with restrictions, they played an important role in serving the State and the authorities in the fight against Covid-19. For example, at the *Christian Ministry of Spiritual Warfare*, a leader explained:

“the role [of the church] was just to keep reminding Christians not to forget that there is a measure that was taken by the government and that Christians must respect”.

The anti-Covid-19 measures were also used by some religious leaders, in particular revivalist leaders, to stress the need to follow the rules—including those of the government–to stay on the right moral and religious path. A Muslim leader developed this idea:

“the main thing is to teach the government to teach people to be serious in life, to fear sin because sin attracts all sorts of diseases”.

At Chrisco, an independent church, the same approach is rephrased in biblical terms:

“The Bible also says that we must know how to render to others what belongs to them and also render to God what is God’s! For we must respect the authorities in our country if the church is to move forward”.

#### Depoliticising discontent

For religious leaders, working to promote the government’s measures typically meant ‘depoliticising’ the issue, showing that combatting the pandemic is not a matter of how much one likes the State, which is far from easy in a context where the disease is only slightly visible and where some in the congregation are in clear opposition to the government. The Comico representative in Bunia explained his strategy, which relies on highlighting how the pandemic affects everyone:

“to convince them [the population/congregation], we say my dear, this [respecting the Covid-19 measures] is not political. Don’t call it politics, it’s too hard; there are already cases when you go to the different hospitals”.

Such ‘depoliticization’ is described as all the more necessary and fragile because of the widespread feeling among leaders and their congregations that the State is not ‘fulfilling its part of the contract’ and has not supported them well enough: “I think the religious [leaders] have done their part, but the government has not organised training” explained a pastor of a Protestant church. The fundamental question raised throughout the interviews was the value of awareness-raising when the means to fight against Covid-19 (screening, treatment, and vaccines) are minimal. Religious leaders were left with an impression of powerlessness, as a CECA representative explained:

All religious denominations are also struggling in this sense [awareness raising]. Because even to relay, to popularise, it is as if we are sending people to hell. You popularise but without really accompanying them in the consequences of the measures.

Most religious leaders considered that they had worked to spread a message that they did not, in fact, fully understand when the (perceived) low prevalence of the virus during the period under study required significant persuasion efforts. The workshop revealed significant misunderstandings about Covid-19, which religious leaders were aware of, as a CCC pastor explained:

We must first clarify [that the government gives quality information to religious leaders] so that people understand the information, the origin of the disease, and the medicines that can cure the disease. [Otherwise] these are confusing things.

The problem seemed more about explaining the logic of the containment and Covid-19 measures than the measures themselves, as handwashing has a direct echo in the scriptures of the Koran and the Bible, as a pastor of an independent church explains:

“Leviticus 13:46: ... This is confinement. He will be confined alone as our pastor Grandpa and his family were confined. ... The others were also affected. If you also read in exodus 30:18–21 you will find there is a problem with hand washing. You have to wash your hands. If you read again Leviticus 13:4–5, it is still the same thing. That is containment. Isaiah 26:20, lockdown always.“

#### Which space for dialogue?

The question the old and new religious actors raise is also about the space for interaction between them and the (health) authorities. From the perspective of the health authorities, there is a general sense of relief that religious denominations helped, but it does not seem to affect the way they envisage future relationships. They stress that the ‘old actors’, the denominations running health facilities, did their work as usual, being as often “more conscientious” than State structures (interview health zone, 2). They do not see a need for change or new structures to accommodate the ‘newcomers’, the denominations that do not run healthcare facilities and came to help during the pandemic. They point that communication can go through existing participatory mechanism such as community health workers and health facility committees. However, the situation from the perspective of religious leaders, and especially the ‘newcomers’, is very different: they talked of their frustration with a system described as unidirectional and stressed that they “cannot really talk” to the authorities. The DRC health system is not, in general, the most open to bottom-up initiatives, and this situation seemed exacerbated in a tense security context, with some interviewees pointing to possible additional tension arising from the “newcomer” religious denominations putting an emphasis on spiritual health, a concept that is alien to a biomedical health worldview.

Coordination structures did exist on paper, mostly through the coordination platforms set up by humanitarian organisations. The most-cited example was the multi-sectoral structure set up with the Ebola response team, which was chaired by a Catholic priest and aimed to create this link but, as one observer of this platform explained: “with Ebola this structure worked very well, because there was a token (cash payment, *jeton de présence*) at each meeting; this is not the case with Covid-19”. The challenge identified through our interviews seems to be for the space to exist without and beyond aid organisations and the financial incentives they introduce.

## Discussion

Our research shows how religious leaders and institutions handled the lockdown period, notably by: (1) interpreting the pandemic and anti-Covid-19 measures within a theological framework which emphasised civic behaviour and the spiritual test of the diseases; (2) using and redeploying structures at the grassroots (the cells); but also (3) responding to the call of the government and health authorities. In an extremely difficult context like Ituri, where conflict is frequent and health interventions are regularly perceived as politicised [14], it is remarkable that leaders of both the more established and less established religious groups generally choose the path of collaboration rather than opposition. Many had significant reservations about the implementation of the anti-Covid-19 measures, resonating with examples of opposition of faith groups and leaders to Covid-19 measures globally [27]. An important distinction needs to be made between religious leaders who express a clear understanding of being subject to the temporal power of the government, and their congregations who are often more willing to be critical—this distinction also points to a potential tension that we have not been able to explore in detail. It would be important to see further studies explore this theme, as it remains possible that this discourse of ‘understanding’ of the State’s approach despite the constraints is partly a veneer.

The religious leaders we interviewed, especially those whose congregations were not previously involved in healthcare provision, also emphasised their awareness of providing a service, or even doing a favour, to the State and the health authorities. This service is mostly at the level of awareness-raising, which is a widely discussed theme in the literature [28]. However, by publicly committing themselves against Covid, religious leaders are participating—and they are aware of this– in an effort to depoliticise Covid-19. It is crucial in the tense context of Ituri. Without going too far into the debate on the often necessarily political nature of health [29], it is useful to note that this depoliticization has two potentially contradictory effects. On the one hand, it allows for the achievement of short-term health objectives such as the reduction of the spread of the epidemic through the respect of health measures. On the other hand, it also limits criticism of the political system and the government, which could, in the longer term, bring health benefits. This is not a new dilemma and is often discussed in humanitarian contexts [30, 31]. The more established denominations, mainly the Catholic Church and to a lesser extent the Anglican Church, are used to this constant balancing act between collaborating with State structures by providing services to fill a gap left by a fragile State and criticizing the government, sometimes vehemently. We see with the Covid-19 crisis in Ituri that other actors, because they are asked to play a health promotion role, find themselves in a similar position. It is, of course, not insignificant that, in the same way that Anglicans and Catholics are conscious of making up for the State’s failures, the ‘newcomer’ denominations in the field of health also express a conviction that they are doing the work that the State should do. There is, of course, more than one possible position. The Catholic Church, for instance, with its large congregation and international support, is both highly critical of the government and involved in State services. The ‘newcomers’ are reviewing their positioning in this space, having been stung by the mobilisation during Covid. Among them, the Pentecostal churches, traditionally separate and critical of the government, responded to President Tshisekedi’s call to participate in the management of the (State) Covid-19 response fund, a call that Anglicans and even Catholics declined [32].

Our analysis shows that the relationship between religious denominations and the State is also at a crossroads in terms of the space for dialogue at the frontline. Platforms set up during the Ebola epidemic did provide some space for inter-faith and inter-sectoral work but they will need to be considerably revamped to survive in the long run, both in terms of resources and incentives to participate. Even more worrying perhaps, is our data suggesting that the frustration of religious leaders—who undoubtedly feel that they have sacrificed a lot– grow as they feel unheard. This needs to be considered seriously by the (health) authorities who should not simply assume that all actors will be happy to revert to the pre-Covid-19 situation.

Our research suffers from obvious limitations, notably the fact that it focuses on urban and peri-urban context and is limited to a small number of participants (mostly religious leaders); these methodological limitations are a consequence of the insecure situation in Ituri, but it is also very clear that other fields are barely touched upon by our research and deserve to be explored further. The reaction of religious congregations to the reconfiguration of the relationship between their denomination and the State and, more generally, the tensions between the views of the population and religious leaders, seem to be a priority for future research, as well an analysis over time of the ongoing reconfiguration.

## Conclusion

The Covid-19 pandemic led to a mobilisation of religious authorities who responded to the authorities’ call to play a role in raising awareness and controlling a population that has very limited trust in the State. In playing such role, they often tried to downplay the political dimension of the pandemic to increase people’s adherence to preventative measures. The mobilisation brought new religious actors close to the health system and, from their perspective, seems to signify a reconfiguration of the relationship between the State (health system) and religious groups. These new actors are putting forward the desire for a relationship that is not just subordination of the religious to the imperatives of health care but a dialogue that allows the experiences of the faithful in conflict zones to be brought to the fore. Recommendations are always hazardous but any way forward requires a space for dialogue that is sensitive to the perspectives of faith groups and relies on long-term trust and commitments rather than short-term humanitarian incentives.

## Data Availability

The datasets (anonymised interview and focus group discussion transcripts) used and/or analysed during the current study are available from the corresponding author on reasonable request.
